# Using Deep Learning to Distinguish Highly Malignant Uveal Melanoma from Benign Choroidal Nevi

**DOI:** 10.3390/jcm13144141

**Published:** 2024-07-16

**Authors:** Laura Hoffmann, Constance B. Runkel, Steffen Künzel, Payam Kabiri, Anne Rübsam, Theresa Bonaventura, Philipp Marquardt, Valentin Haas, Nathalie Biniaminov, Sergey Biniaminov, Antonia M. Joussen, Oliver Zeitz

**Affiliations:** 1 Department of Ophthalmology, Charité University Hospital Berlin, 12203 Berlin, Germany; 2 HS Analysis GmbH, 76131 Karlsruhe, Germany

**Keywords:** deep learning, artificial intelligence, choroidal melanoma, fundus imaging

## Abstract

**Background:** This study aimed to evaluate the potential of human–machine interaction (HMI) in a deep learning software for discerning the malignancy of choroidal melanocytic lesions based on fundus photographs. **Methods:** The study enrolled individuals diagnosed with a choroidal melanocytic lesion at a tertiary clinic between 2011 and 2023, resulting in a cohort of 762 eligible cases. A deep learning-based assistant integrated into the software underwent training using a dataset comprising 762 color fundus photographs (CFPs) of choroidal lesions captured by various fundus cameras. The dataset was categorized into benign nevi, untreated choroidal melanomas, and irradiated choroidal melanomas. The reference standard for evaluation was established by retinal specialists using multimodal imaging. Trinary and binary models were trained, and their classification performance was evaluated on a test set consisting of 100 independent images. The discriminative performance of deep learning models was evaluated based on accuracy, recall, and specificity. **Results:** The final accuracy rates on the independent test set for multi-class and binary (benign vs. malignant) classification were 84.8% and 90.9%, respectively. Recall and specificity ranged from 0.85 to 0.90 and 0.91 to 0.92, respectively. The mean area under the curve (AUC) values were 0.96 and 0.99, respectively. Optimal discriminative performance was observed in binary classification with the incorporation of a single imaging modality, achieving an accuracy of 95.8%. **Conclusions:** The deep learning models demonstrated commendable performance in distinguishing the malignancy of choroidal lesions. The software exhibits promise for resource-efficient and cost-effective pre-stratification.

## 1. Introduction

Choroidal nevi, prevalent in 4–20% of the Caucasian population, stand as the most common benign intraocular tumors [1]. Conversely, malignant choroidal melanomas are rare, occurring at an incidence of 5–6 per million inhabitants. Assuming that all choroidal melanomas originate from transformed choroidal nevi, the estimated malignant transformation rate is approximately 1 in 8845 [2]. Regular follow-up examinations, spaced between 3 months and 2 years, are recommended based on the appearance of these lesions. However, in outpatient settings, the assessment of the malignancy potential of choroidal lesions is often constrained. Due to the low prevalence of uveal melanoma, most primary eye care providers rarely encounter this entity, leading to the referral of patients with risk factors to specialized ocular oncology departments.

Multimodal imaging has identified risk factors for the transformation of a nevus into a melanoma, including thickness, subretinal fluid, orange pigment, and melanoma hollow [3,4,5]. In a longitudinal study, 13.9% of choroidal nevi had transformed to melanoma in 10 years, whereas the transformation rate increased with the number of imaging risk factors [4]. The MOLES acronym comprises clinical features such as mushroom shape, orange pigment, large size, enlargement, and subretinal fluid to assist non-experts in the evaluation of malignancy [6]. Lesions are referred to as low- or high-risk nevi or possible melanoma according to the sum of the score. Regular follow-up examinations are commonly administered by specialized tumor centers equipped with multimodal imaging capabilities. In contrast to these technologies, color fundus photographs (CFPs) are generally widely available in outpatient settings and optometrists’ offices. Improving screening techniques is essential to increase globe salvage and preserve useful vision. Moreover, timely diagnosis is crucial given the poor efficacy of metastatic disease treatment [7,8].

Ophthalmic diseases have significant potential for telemedicine and deep learning applications due to their increasing reliance on image-based investigations [9]. Deep learning is a subset of machine learning involving the processing of data in multiple layers to progressively extract higher-level features from the raw input [10]. These models have led to increased efficiency in the recognition of subtle patterns in complex medical data formats [11]. In ophthalmic diseases, deep learning applications include the segmentation of the optic disc and blood vessels, the detection of lesions, classification, and the prediction of disease progression in age-related macular degeneration and diabetic retinopathy [12,13]. In order to improve accessibility and limit costs, several studies have successfully used deep learning algorithms to screen for pathologies in CFPs, such as myopic maculopathy, peripheral retinal degenerations, diabetic retinopathy, and glaucomatous disc changes [14,15,16]. A deep learning algorithm achieved high accuracy in distinguishing active retinoblastoma from normal fundus images or stable disease [17]. Furthermore, the retinoblastoma screening tool was cost-effective compared to existing procedures based on referral to specialized centers.

While morphological factors in multimodal imaging indicative of choroidal lesion malignancy have been established in studies, to our knowledge, none have employed deep learning algorithms for assessing their malignancy. Automated image analysis offers a potential avenue for efficiently differentiating the malignancy of choroidal lesions, providing resource- and cost-effective pre-stratification based on color fundus photography.

Therefore, this study seeks to explore the feasibility of an artificial intelligence-based assistant to employ deep learning models for the classification of the malignancy of melanocytic choroidal lesions. Additionally, we compare various deep learning architectures for binary and multi-class classification tasks [18,19,20,21]. If applicable in clinical practice, the integration of automated image analysis software holds the potential to provide referral advice and streamline the care reality for affected patients.

## 2. Materials and Methods

This study received approval from the Institutional Review Board at Charité University Hospital Berlin and adhered to the principles of the Declaration of Helsinki. The dataset comprised 762 consecutive color fundus photographs (CFPs) retrospectively collected from subjects seen at the Ocular Oncology Department of Charité University Hospital Berlin between January 2010 and January 2023. The inclusion criteria covered subjects diagnosed with choroidal nevus, treatment-naïve choroidal melanoma, and irradiated choroidal melanoma, with irradiation involving either proton beam therapy or plaque radiotherapy. The exclusion criteria included inadequate imaging quality, prior treatment at another clinic, and prior endoresection surgery.

All subjects underwent multimodal imaging, including biomicroscopic examination, spectral domain optical coherence tomography (OCT) imaging, fundus autofluorescence, and ultrasonography. Retinal specialists initially classified subjects based on multimodal imaging findings, with malignancy criteria encompassing thickness exceeding 2 mm (via ultrasonography), subretinal fluid on OCT, presence of symptoms, orange pigment (via autofluorescence), melanoma hollow and low internal reflectivity (via ultrasonography), and basal extension exceeding 5 mm on photography. The consensus identification served as the reference for training deep learning models, and the data were split into 90% for training and 10% for internal validation. Subsequently, deep learning models were tested on an independent test set comprising images not included in the training and internal validation set. Furthermore, the MOLES score classifying the lesions based on mushroom shape, orange pigment, large size, enlargement, and subretinal fluid was calculated for the independent test set. Tumors were considered as common nevi, low-risk nevi, high-risk nevi, or probable melanoma in case of a sum of these scores of 0, 1, 2, or 3 and more. Subsequently, we compared the classification of the test images based on the MOLES score and the deep learning models.

**Figure 1:**
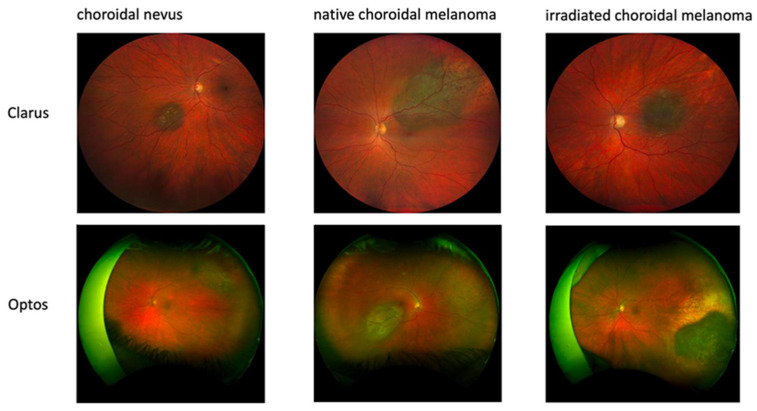
Examples of training data acquired with different imaging techniques.

Color fundus images were acquired using either wild-field Optos (Daytona, Optos PLC, Dunfermline, UK) or Clarus (ZEISS, Jena, Germany) devices based on the choroidal lesion’s location (Figure 1).

**Figure 2:**
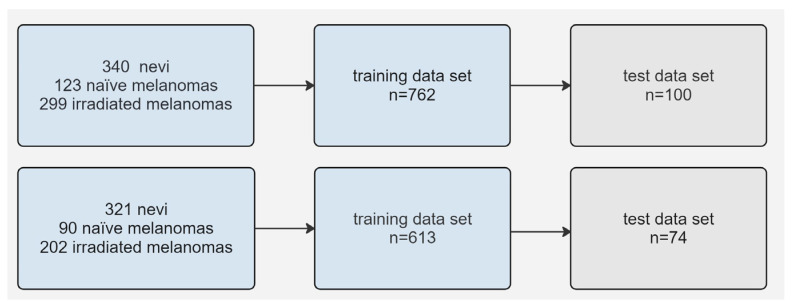
Flow diagrams of the image datasets used for trinary classification; the set represented by the above boxes included Optos and Clarus images, and that represented by the below boxes solely included Clarus images.

We created four distinct models using 762 images obtained by Clarus and Optos imaging and 613 images exclusively obtained by Clarus imaging (Figure 2). Furthermore, we created binary classification models for both settings including only choroidal nevi and naïve choroidal melanomas.

Each CFP was saved as a JPEG file. In medical applications, the use of convolutional neural networks (CNNs) has proven to be a robust approach. For example, Unet is still widely used in cell segmentation and classification [22]. Although transformers have achieved significant breakthroughs in recent years, their lack of inductive bias requires an enormous amount of data. Therefore, in our study design, we employed a CNN due to the limitation in relation to the amount of training data in regard to the low prevalence of the disease. A convolutional neural network (CNN) architecture named HyperTumorEyeNet, based on ResNet50 [23], was employed using the software HSA KIT version 1.5.13.10 for trinary (nevus vs. treatment-naïve melanoma vs. irradiated melanoma) and binary (nevus vs. treatment-naïve melanoma) classifications. The standard ResNet50 model was initialized using pretrained weights provided by the HSA KIT. The pretrained weights were created by training the ResNet50 model in a supervised manner on a variety of medical classification tasks. Besides the ResNet50 model, we additionally compared HyperTumorEyeNet based on EfficientNet B4, Vision Transformer initialized with Segment Anything weights (due to the positional embedding, an image size of 224 was used), and, finally, a ConvNextV2 base model [18,19,20,21]. The comparison was conducted using the training set of Optos and Clarus images (n = 762). To reduce the required training time, the evaluation was performed on a subset of the test set specific to this category. Subsequently, the best-performing model was selected for training and evaluation across all classification tasks (Table 1).

**Figure 3:**
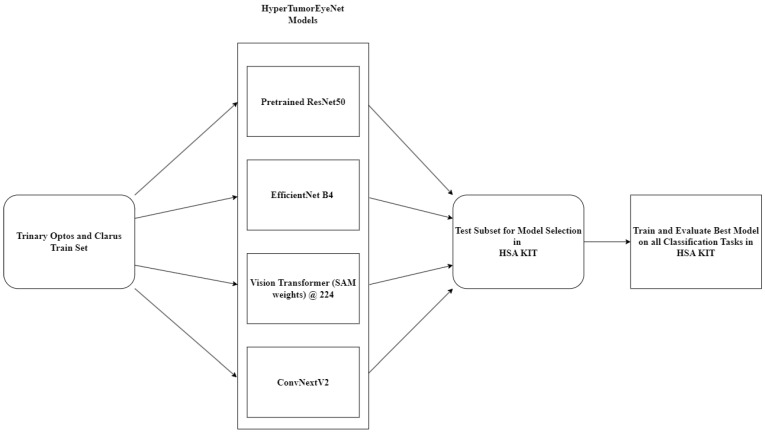
Flowchart of the model selection process.

The model selection process is illustrated in Figure 3.

**Figure 4:**
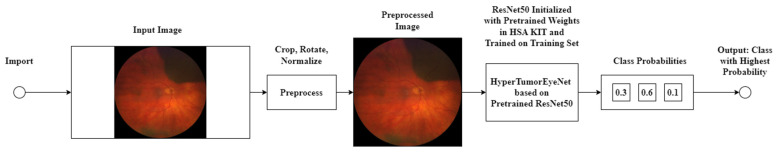
Inference flowchart showing the predicted class probabilities.

The models underwent 100 epochs, incorporating preprocessing steps such as cropping the region of interest due to there being a white border around it, random rotation, horizontal and vertical flipping, and normalization and validation, as depicted in Figure 4, and these steps were conducted by the preprocessing box. The output of the model is highlighted in the class probabilities box, which shows the predicted class probabilities for the given input image (in this case, 0.3 for treatment-naïve melanoma, 0.6 for nevus, and 0.1 for irradiated melanoma. All models were trained using the Adam optimizer with a learning rate of 0.0001 and a batch size of 16.

The training regimen incorporated early stopping, capping at 100 epochs; thus, training was halted if no improvement was discernible within this limit. Notably, a collaborative approach between human experts and machine learning was adopted, facilitated by the iterative process within HSA KIT. This iterative framework enabled continual refinement and optimization of the model through successive cycles of training, evaluation, and feedback. Throughout training and validation, diverse data augmentation techniques were employed, including random flipping along multiple axes, Gaussian blur application, and affine transformations. Model performance was evaluated using accuracy, precision, recall (sensitivity), specificity, and F1 score.

Testing was conducted on a set of 100 independent images for the trinary classification and 74 independent images for the binary classification, with the images consisting of nevi, treatment-naïve melanomas, and irradiated melanomas. Since the models performed multi-class classification, a one-vs-all approach was employed to calculate precision, recall, specificity, and F1 score for each individual class. This means that for each class, the metrics were calculated by considering the class of interest as the positive class and all other classes combined as the negative class. Conversely, accuracy was computed by considering all classes collectively, furnishing a holistic evaluation of the model’s performance across all categories. Subsequently, we evaluated models of trinary and binary classification upon the exclusion of Optos images in both the training and test sets. Confusion matrices were drawn for each model, cross-tabulating ground truth labels versus the labels classified by the deep learning models.

Statistical analysis involved calculating the area under the receiver operating characteristic curve (ROCs) using both micro- and macro-averaging techniques. Micro-averaging considers the entire dataset as a single large set, giving equal weight to each sample, while macro-averaging calculates the metrics for each class independently and then takes the unweighted mean of those metrics. The use of both averaging methods provides a comprehensive view of the model’s performance in the multi-class setting. Descriptive data are presented as mean or percentages.

## 3. Results

The initial model comparison was carried out on a subset of the multi-class classification task based on Optos and Clarus images. Table 1 shows the performance of the compared architectures. ResNet50 achieved the highest performance, with an accuracy of 92.65%, followed by EfficientNet B4, which had an accuracy of 86.67%, Vision Transformer initialized with Segment Anything weights, which had an accuracy of 79.41% (using an image size of 224 due to positional embedding), and the ConvNextV2 Base model, which had an accuracy of 77.94%. The superior performance of ResNet50 is most likely due to the good pretraining weights provided for the model and the relatively small dataset, which makes advanced methods less effective. Accuracy is a suitable performance metric here since the dataset is completely balanced. Based on these results, ResNet50 was chosen for further evaluation, and in this paper, it is from now on referred to as “the model” for simplicity.

Models involving both Optos and Clarus images were tested on an independent test set of 100 images for trinary classification (33 choroidal nevi, 33 naïve choroidal melanomas, and 34 irradiated choroidal melanomas) and 66 images (33 nevi and 33 naïve choroidal melanomas) for binary classification (benign vs. malignant). Overall, binary classification of malignancy showed improved discriminative performance compared to trinary classification. Table 2 shows the averaged discriminative performance of all models.

**Figure 5:**
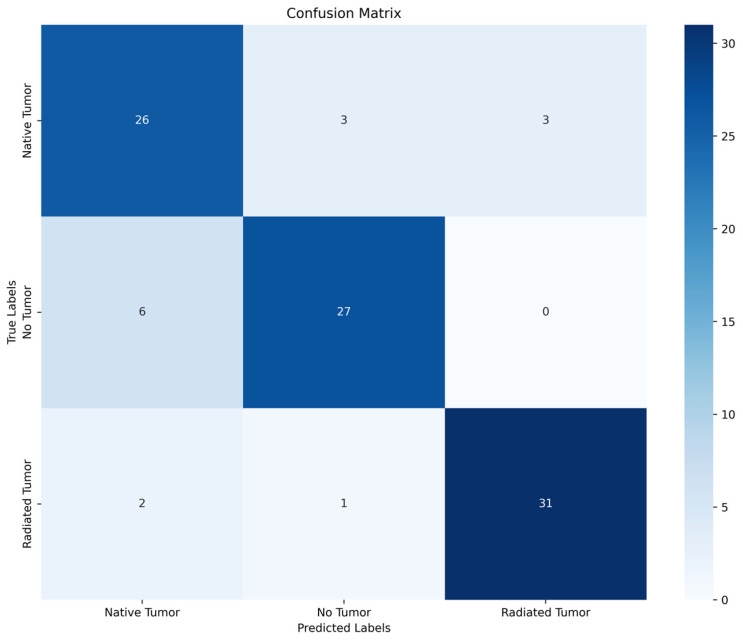
Confusion matrix of trinary classification model of Optos and Clarus images.

The mean accuracy values for all classes were 84.8% for trinary classification and 90.9% for binary classification. Regarding all three classes, the highest accuracy was achieved for the classification of irradiated choroidal melanoma (94.0%). The mean precision, recall, specificity, and F1 score values across all three categories were recorded as being 0.85, 0.85, 0.91, and 0.85, respectively. The confusion matrix presented in Figure 5 indicates categories in which the model performs insufficiently.

The results indicate that 9.1% of naïve choroidal melanomas were misclassified as either irradiated choroidal melanomas and nevi. Furthermore, 18.1% of nevi were classified as naïve choroidal melanomas.

**Figure 6:**
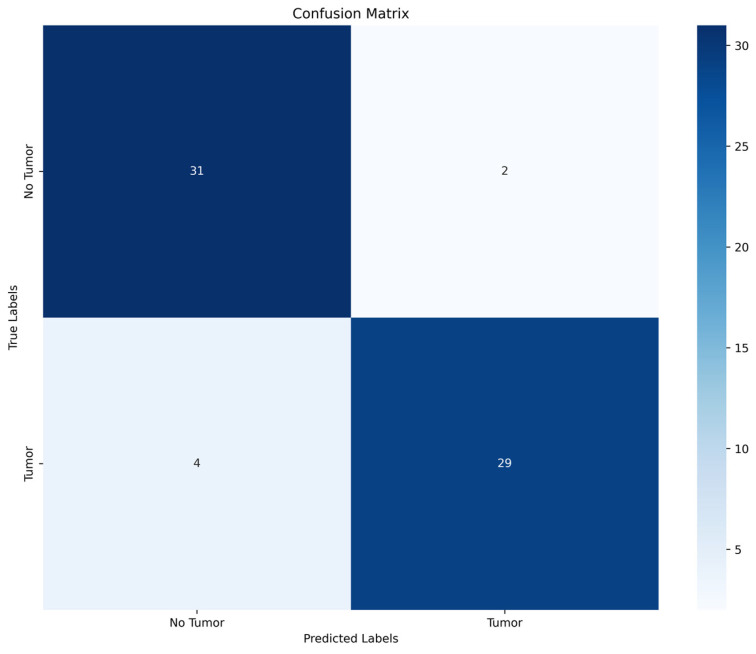
Confusion matrix of binary classification of Optos and Clarus images.

Subsequently, we evaluated binary classification performance (benign vs. malignant) in order to investigate discriminative performance as a screening tool. Table 1 displays improved accuracy (90.9%) and recall (0.90) compared to trinary classification performance. The confusion matrix of binary classification (Figure 6) indicates that 12.1% of malignant lesions were misclassified as benign.

**Figure 7:**
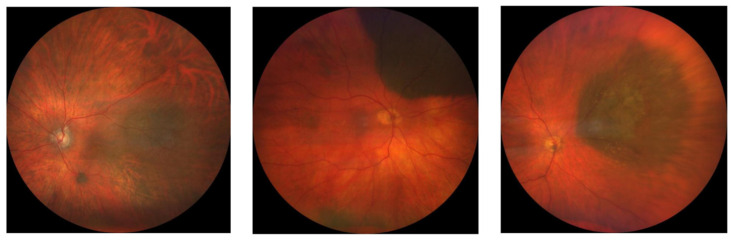
Examples of choroidal melanomas misclassified as choroidal nevi by both models.

In a real-world setting, this would lead to less urgent referral and possibly delayed diagnosis and treatment. Overall, 6.1% of choroidal nevi were classified as malignant lesions resulting in earlier referral than potentially needed. The test results indicated that three naïve choroidal melanomas were misclassified as choroidal nevi by both binary and trinary classification (Figure 7). Upon comparison with multimodal imaging features of these lesions, all three choroidal lesions exhibited moderate thicknesses of below 3 mm and moderate internal reflectivity on ultrasonography. Given these borderline clinical features, assessment of their malignancy relied on several multimodal imaging findings. To determine their malignancy, one of the lesions underwent histopathologic confirmation before treatment, and one lesion was classified as a transformed choroidal nevus. Furthermore, one different naïve choroidal melanoma was misclassified as choroidal nevi by one model.

Subsequently, we evaluated models of trinary and binary classification upon the exclusion of Optos images in both the training and test sets. The results indicate improved discriminative performance in multi-class and binary classification. The mean accuracy in the multi-class problem was 86.5%, compared to 84.8% when including different imaging techniques. The highest performance was achieved in binary classification, with an accuracy of 95.8% and recall of 0.95. The confusion matrices reveal that in binary classification, all choroidal nevi were correctly classified, and 10% of naïve choroidal melanomas were misclassified as benign.

**Figure 8:**
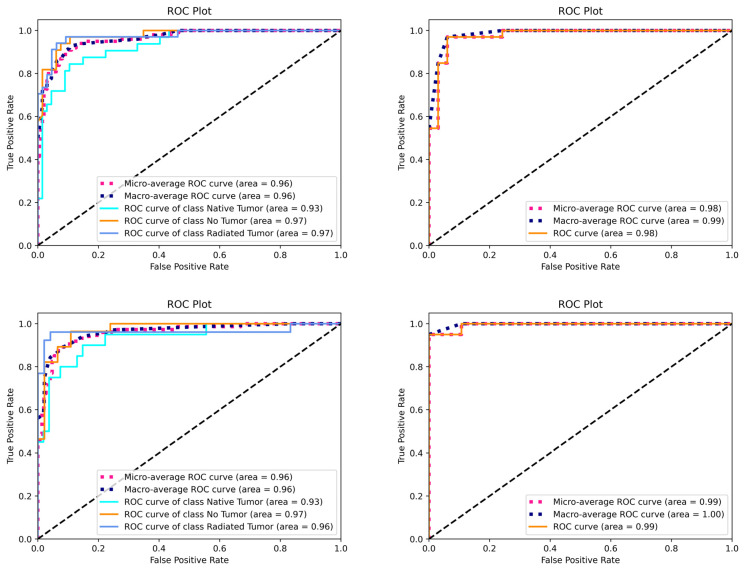
ROC curves of all models for trinary and binary classification of Optos and Clarus images (**above**) and for trinary and binary classification based on Clarus images (**below**).

The mean area under the curve (AUC) of the receiver operating characteristic curves (ROCs) of all models is shown in Figure 8. The ROC analysis demonstrates high averaged AUC values ranging from 0 to 1.00, with the best diagnostic performance being for binary classification based on Clarus images.

We calculated the MOLES score for the test set consisting of choroidal nevi and naïve choroidal melanoma, which serves as a tool to determine the likelihood of malignancy in choroidal tumors (Table 3). The lesions were estimated as common nevi, low-risk nevi, high-risk nevi, and probable melanoma according to the sum of the subscores of 0, 1, 2, or 3 and more, respectively. Overall, the deep learning-based classification was slightly inferior to the MOLES score. All 33 tumors in the test set diagnosed as naïve uveal melanoma by the retinal experts had a MOLES score of 3 or more, whereas the deep learning model classified 4 out of these 33 tumors as benign (12.1%). Overall, 2 out of 33 lesions diagnosed as nevi by retinal experts had a score of 3, indicating probable melanoma. The deep learning model classified one of them as choroidal melanoma and one as a nevus.

## 4. Discussion

In this proof-of-concept study, we developed several deep learning models through human–machine interaction (HMI) implemented in HSA KIT software for the classification of the malignancy of choroidal lesions based on CFPs. Discriminative performance was improved in binary classification compared to trinary classification. The best performance was achieved upon using images of a single imaging modality. To the best of our knowledge, this is the first study to evaluate the deep learning-based classification of choroidal tumors where the acquisition of a robust dataset is challenging due to their low prevalence [24].

Fundus imaging is a widely available tool in outpatient settings, whereas multimodal imaging is often reserved for specialized clinics. Therefore, the development of a software based on CFPs could lead to resource- and cost-effective pre-stratification. The deep learning models based on a single imaging technique reached an accuracy that was only slightly inferior to the MOLES score, which relies on multimodal imaging. Early detection of malignant disease is crucial since treatment of metastatic disease is only rarely effective [25]. On the other hand, immediate treatment of indeterminate lesions is associated with potential vision loss [26]. Hence, there is a need to improve the accuracy of non-invasive diagnostics considering the possible complications of pathologic confirmation (including the seeding of tumor cells and iatrogenic retinal detachment and vitreous hemorrhage) [27,28].

Recent studies showed the feasibility of the deep learning-based detection of various retinal pathologies, such as peripheral retinal degenerations, using wide-field fundus imaging [16]. Furthermore, deep learning algorithms have been shown to detect diabetic retinopathy and diabetic macular edema in fundus photographs [12].

In the absence of studies evaluating deep learning in choroidal tumors, deep learning-based models showed satisfying performance in the classification of tumors of various organs, including lung, skin, and orbital tumors [29,30,31]. Wei et al. developed a deep learning assistant for retinoblastoma monitoring [17]. The authors found comparable discriminative performance, as in our study. The algorithm achieved an area under curve (AUC) of 0.99 in distinguishing normal fundus and active retinoblastoma and a value of 0.94 in distinguishing stable and active retinoblastoma. The diagnosis accuracy and sensitivity were non-inferior to ophthalmologists with 2–5 years of experience in examination under anesthesia.

Faes et al. analyzed an automated deep learning software for medical image diagnostic classification by healthcare professionals with no coding or deep learning expertise, meaning their study is comparable to our study [32]. They described comparable discriminative performance from internal validations in binary classification tasks (recall 73.3–97.0%; precision 67–100%; AUPRC 0.87–1.00). Considering multiple classification, the diagnostic properties ranged from 38% to 100% for recall, from 67% to 100% for precision, and from 0.57 to 1.00 for AUPRC.

In this study, we reviewed the misclassified cases and compared them to the corresponding multimodal imaging findings. Three choroidal melanomas were misclassified as choroidal nevi by both the trinary and binary models. These exhibited findings such as borderline thickness or moderate internal reflectivity on ultrasonography. In some cases, the final classification of the retinal specialists, serving as the reference standard, included lesion growth during follow-up or histopathologic confirmation. Furthermore, we compared the discriminative performance to the MOLES score, which was designed to help non-experts in the assessment of malignancy. We found the deep learning algorithm to be commendable but slightly inferior to the MOLES score in the classification of uveal melanoma, whereby discordance between the diagnosis of retinal experts and the deep learning model was mainly seen in borderline cases with a score indicating probable melanoma. Overall, due to the lack of consensus amongst ocular oncologists as to which suspicious tumors should be considered malignant, a definitive classification remains infeasible in the absence of histopathologic confirmation.

All models exhibited commendable recall, which was improved in binary classification compared to multi-class classification. A deep learning model for screening purposes in tumor classification would ideally exhibit a high sensitivity in order to prevent delayed diagnosis and treatment. In our study, the naïve choroidal melanomas were mainly falsely classified as irradiated choroidal melanomas. We suppose that morphologic changes after irradiation are more subtle than distinction with benign lesions. Furthermore, they depend on the time elapsed and mode of treatment.

Our proof-of-concept study has several limitations. The study’s development was based on a relatively small dataset that was not validated on an external dataset. Given the rarity of choroidal melanomas in the overall population, the acquisition of a greater number of imaging samples was hindered. In this study, two different fundus imaging technologies were included in two models depending on the location of the lesions. In contrast to pseudocolor imaging, used in Optos, the Clarus device offers true color imaging, therefore increasing the variability among the images. On the other hand, the inclusion of pseudocolor fundus imaging has the potential to extend the algorithm to optometry offices, where they are often employed as a screening tool. Furthermore, the addition of a supplementary imaging modality such as OCT, with widespread use in outpatient settings, could lead to improved discriminative performance.

Furthermore, although the reference standard was set by retinal specialists based on multimodal imaging, final diagnosis of malignancy of a choroidal lesion can only be made on histological findings. Since the biopsy of a choroidal lesion involves the risk of multiple complications, including the seeding of tumor cells, it is not routinely used in clinical practice. Therefore, the included data may as well involve indeterminate lesions whose appropriate management is debated. The composition of the images of irradiated melanomas might have been heterogeneous, considering the different radiation methods.

Incorporating recent advancements into deep learning, such as attention mechanisms and multi-scale feature extraction, could potentially enhance the analysis of choroidal lesions. Self-attention or channel attention can guide the model to focus on the most relevant regions of the image for classification, while techniques like feature pyramid networks or atrous spatial pyramid pooling can capture both local and global contexts, improving the model’s ability to handle lesions of varying sizes and shapes.

The acquisition of a larger dataset is crucial for improving the model’s performance and generalization capability. However, data scarcity poses a significant challenge in medical image analysis due to privacy concerns, limited resources, and the rarity of certain conditions, such as choroidal melanomas. Collaborative efforts among multiple institutions to share and pool data could help alleviate this issue. Additionally, data augmentation techniques, including elastic deformations, color jittering, and random cropping, can artificially increase the diversity of the training data.

Given the nature of the medical domain, online learning could be a valuable approach for continuously improving the model’s performance as new data become available. This approach allows the model to adapt and learn from new examples without the need to retrain the entire model from scratch, which is particularly useful when the distribution of data may shift over time or when new subtypes of lesions are encountered.

Further work could involve visual evaluation techniques, such as Gradient-weighted Class Activation Mapping (Grad-CAM), to provide interpretability and transparency to the model’s predictions. Grad-CAM highlights the regions of the image that contribute most to the model’s decision, allowing clinicians to understand the basis of the model’s classification.

In this pilot study, we evaluated choroidal lesion images obtained at a single institution. Further studies should include external prospective validation images across all imaging devices and patient populations.

## 5. Conclusions

To conclude, we report that the aforementioned deep learning models have the ability to assess malignancy of choroidal lesions with satisfying discriminative performance compared to experienced retinal specialists. Further studies need to be conducted to determine the feasibility of applying these algorithms in a clinical setting and allow for resource-efficient pre-stratification.

## Figures and Tables

**Table 1:** Performance in multi-class classification of different architectures reported on a completely balanced dataset.

Deep Learning Model	Accuracy
ResNet50	92.65%
EfficientNet B4	86.67%
Vision Transformer (SAM weights)	79.41%
ConvNext Base	77.94%

**Table 2:** Averaged discriminative performance of all models.

Deep Learning Model	Accuracy	Precision	Specificity	Recall	F1 Score	AUC-ROC
Trinary classification Optos and Clarus images	84.8%	0.85	0.92	0.85	0.85	0.96
binary classification Optos and Clarus images	90.9%	0.91	0.91	0.90	0.91	0.99
trinary classification Clarus images	86.5%	0.87	0.93	0.85	0.85	0.96
binary classification Clarus images	95.8%	0.97	0.95	0.95	0.96	1.00

**Table 3:** Classification of test images by the deep learning model and the experts according to the MOLES score.

MOLES Score	Classified as Nevi by Deep Learning, Total n (%)	Classified as Choroidal Melanoma by Deep Learning, Total n (%)	Classified as Nevi by Retinal Experts, Total n (%)	Classified as Choroidal Melanoma by Retinal Experts, Total n (%)
0 (n = 15)	15 (100)	0	15 (100)	0
1 (n = 12)	11 (91.7)	1 (8.3)	12 (100)	0
2 (n = 3)	3 (100)	0	3 (100)	0
3 (n = 2)	1 (50)	1 (50)	2 (100)	0
>3 (n = 33)	4 (12.1)	29 (87.9)	0	33 (100)

## Data Availability

The data presented in this study are only available on request from the corresponding author due to privacy restrictions.
